# Aging in Normotensive and Spontaneously Hypertensive Rats: Focus on Erythrocyte Properties

**DOI:** 10.3390/biology12071030

**Published:** 2023-07-21

**Authors:** Jana Radosinska, Marta Kollarova, Tomas Jasenovec, Dominika Radosinska, Norbert Vrbjar, Peter Balis, Angelika Puzserova

**Affiliations:** 1 Institute of Physiology, Faculty of Medicine, Comenius University in Bratislava, Sasinkova 2, 811 08 Bratislava, Slovakia; marta.husseinova@fmed.uniba.sk (M.K.); tomas.jasenovec@fmed.uniba.sk (T.J.); 2 Centre of Experimental Medicine, Slovak Academy of Sciences, Dúbravská Cesta 9, 841 04 Bratislava, Slovakia; norbert.vrbjar@savba.sk (N.V.); peter.balis@savba.sk (P.B.); angelika.puzserova@savba.sk (A.P.); 3 Premedix Academy, Medená 18, 811 02 Bratislava, Slovakia; 4 Institute of Medical Biology, Genetics and Clinical Genetics, Faculty of Medicine, Comenius University in Bratislava, Sasinkova 4, 811 08 Bratislava, Slovakia; dominika.radosinska@fmed.uniba.sk

**Keywords:** hypertension, erythrocyte deformability, blood pressure, nitric oxide, spontaneously hypertensive rats, aging, oxidative stress

## Abstract

**Simple Summary:**

We focused on changes in erythrocyte parameters—deformability, mean cell volume, and nitric oxide (NO) production by erythrocytes—in spontaneously hypertensive rats (SHR) and normotensive Wistar-Kyoto (WKY) rats, and on possible differences in these parameters as a function of age. We wanted to contribute to a more comprehensive understanding of the widely used animal model of essential hypertension—SHRs. We found that erythrocyte deformability was inversely proportional to the values of systolic blood pressure in normotensive animals, not in hypertensive ones. Regarding the relationship between the size of erythrocytes and blood pressure value, a significant negative correlation was found in SHRs, but not in WKY rats. In both normotensive and hypertensive animals, we observed a direct relationship between erythrocyte deformability and NO production by erythrocytes. Our study also suggests that age-related changes in erythrocyte deformability in both WKY and SHR are at least partially associated with changes in NO production. However, changes in NO production by erythrocytes in hypertension are probably not the primary factor affecting erythrocyte deformability. Summarizing the findings, the interpretation of the data obtained in erythrocyte parameters observed in SHRs to human hypertension needs precaution.

**Abstract:**

Erythrocyte deformability, crucial for oxygen delivery to tissues, plays an important role in the etiology of various diseases. As the factor maintaining the erythrocyte deformability, nitric oxide (NO) has been identified. Reduced NO bioavailability also plays a role in the pathogenesis of hypertension. Our aim was to determine whether aging and hypertension affect erythrocyte deformability and NO production by erythrocytes in experimental animals divided into six groups according to age (7, 20 and 52 weeks), labeled WKY-7, WKY-20 and WKY-52 for normotensive Wistar-Kyoto (WKY) rats, and SHR-7, SHR-20 and SHR-52 for spontaneously hypertensive rats (SHR). The filtration method for the determination of erythrocyte deformability and the fluorescent probe DAF-2 DA for NO production were applied. Deformability and NO production by erythrocytes increased at a younger age, while a decrease in both parameters was observed at an older age. Strain-related differences in deformability were observed at 7 and 52 weeks of age. SHR-7 had reduced deformability and SHR-52 had increased deformability compared with age-matched WKY. Changes in NO production under hypertensive conditions are an unlikely primary factor affecting erythrocyte deformability, whereas age-related changes in deformability are at least partially associated with changes in NO production. However, an interpretation of data obtained in erythrocyte parameters observed in SHRs of human hypertension requires precaution.

## 1. Introduction

Normal red blood cells (RBCs) are biconcave discs. Such a shape offers an increased surface area-to-volume ratio compared to a spherical shape that promotes the diffusion of respiratory gases through the membrane. The discoid shape is supported by peripheral and integral proteins localized in RBC membranes that together form the cytoskeleton of RBCs. The cytoskeleton provides enhanced stability and contributes to the structural deformability of RBCs. This property allows them to pass through capillaries that are narrower than the diameter of RBCs. It is crucial for the quality of microcirculation and the delivery of oxygen to all tissues within the human body. At the same time, the deformability of RBCs is important for their survival within the circulation and responsible for the decrease in blood viscosity, especially at higher shear rates [1]. Reduced deformability of RBCs plays a significant role in the etiology of various diseases including cardiovascular, and also during physiological aging [2,3].

The pathophysiology of cardiovascular diseases is of high importance and connected to impairment in the oxygen-transport function of blood and, in many cases, to the reduction in the efficiency of oxygen transport carried out by RBCs. It was documented that hypertension is connected with impairment of RBC deformability either in humans [4,5,6] or in animal models [6,7]. RBC deformability lowered with an increase in the severity of hypertension [8,9] as well as the duration of hypertension [10].

Concerning factors affecting RBC deformability, nitric oxide (NO) has been identified [11]. RBCs are exposed to the NO produced by other cells, e.g., endothelial, but they are also capable of synthetizing their own NO [12]. Notably, NO bioavailability plays an important role in the pathogenesis of hypertension [13] and also during aging [14]. In addition, both conditions—hypertension and aging—are associated with oxidative stress [15].

A spontaneously hypertensive rat (SHR) represents an inbred rodent model mimicking human essential hypertension. SHRs develop hypertension endogenously with age. In comparison with their normotensive counterparts—Wistar-Kyoto rats (WKY)—blood pressure began to rise in SHR strain at 4 weeks of age [16]. SHR is also a widely and extensively studied animal model of genetic hypertension [17]. Furthermore, SHR serves as a useful model of attention-deficit/hyperactivity disorder [18]. It was shown that improvement of RBC deformability and increased NO bioavailability reduces behavioral hyperactivity in juvenescent SHRs [19]. Thus, we can presume that the impairment of RBC properties may at least partially contribute to clinical symptoms in SHRs.

Taking into consideration all abovementioned theories, we aimed to investigate changes in RBC parameters—deformability, mean cell volume (MCV) and NO production by RBCs—together with characterization of oxidative stress in both SHR and WKY, and to map possible differences in these parameters as a function of age. Such knowledge could contribute to a more comprehensive understanding of the model of essential hypertension, SHR, and help to discuss the results obtained by the use of this model.

## 2. Materials and Methods

### 2.1. Ethical Approval

The study was conducted in accordance with the Declaration of Helsinki, and approved by the State Veterinary and Food Administration of the Slovak Republic (decision No. Ro-3095/14-221 and decision No. Ro-1087/17-221) and by an Ethics Committee (project code: EK/vekhyp/2014, approved 24 June 2014 and EK/1/17, approved 6 February 2017) according to the European Convention for the Protection of Vertebrate Animals used for Experimental and other Scientific Purposes, Directive 2010/63/EU of the European Parliament.

### 2.2. Experimental Animals

Experiments were performed on male SHRs (sublines SHR/NHsd, HARLAN UK) and normotensive WKY rats (sublines WKY/NHsd, HARLAN UK). All rats were born in an accredited establishment at the Institute of Normal and Pathological Physiology, Centre of Experimental Medicine, Slovak Academy of Sciences, to keep an equal environmental background. This study represents part of a more complex experiment with already published details of the experimental protocol and some procedures previously [20,21]. Briefly, animals were housed from 2 to 4 per cage at constant conditions, i.e., temperature (22–24 °C) and humidity (45–60%), 12 h light–dark cycle (light phase from 06:00 to 18:00) and freely accessible standard laboratory rat diet and drinking water. According to age (7 weeks or young juvenile, 20 weeks or young adult and 52 weeks or older rats) and strain (WKY or SHR), the rats were divided into the following six groups: WKY-7, WKY-20, WKY-52 and SHR-7, SHR-20, SHR-52 (*n* = 5–12). General biometric and cardiovascular parameters were monitored: body weight (BW), heart left ventricle weight (LVW), heart rate (HR) and systolic blood pressure (BP) as described previously [20,21]. Trunk blood was collected in heparinized test tubes, while a portion of whole blood (10 μL) was taken to evaluate NO production by RBCs. The rest of the blood was immediately centrifuged (4 °C, 850× *g*, 10 min), RBCs were used for the determination of their deformability and volume. Blood plasma was stored at −80 °C till the biochemical analyses.

### 2.3. Biochemical Analysis of Blood Plasma

The concentration of the N-terminal fragment of pro-B-type natriuretic peptide (NT-proBNP) was evaluated by the use of Rat NT-proBNP ELISA Kit (Elabscience Biotechnology Inc., Houston, TX, USA), according to the manufacturer’s instructions.

The levels of total cholesterol, triglycerides, uric acid and bilirubin were determined in the accredited veterinary laboratory Laboklin GMBH (Bratislava, Slovakia) by the use of standard laboratory methods.

The following markers of oxidative damage were measured: thiobarbituric acid reactive substances (TBARS), a marker of lipid peroxidation; advanced oxidation protein products (AOPP), a marker of protein oxidation; advanced glycation end products (AGEs); and fructosamine, markers of carbonyl stress. As parameters of antioxidant status, we applied the measurement of ferric-reducing antioxidant power (FRAP) and total antioxidant capacity (TAC). In addition, the ratio of reduced oxidized glutathione (GSH/GSSG) was measured as a general marker of oxidative stress. We followed previously published protocols [20,22,23].

### 2.4. Erythrocyte Parameters

As an indicator of NO production by RBCs, a fluorescent probe 5,6-diaminofluorescein diacetate (DAF-2 DA) was used as described previously [24]. Briefly, whole blood was diluted 1:9 in a modified physiological salt solution and incubated with DAF-2 DA (30 μmol/L) for 10 min at room temperature in the dark. Blood samples were analyzed by fluorescence microscope (Nikon Eclipse Ti, Tokyo, Japan) using the filter for the fluorescein isothiocyanate (λ_ex_. 465–495 nm, λ_em_. 515–555 nm). The fluorescent signal was quantified using the ImageJ 1.53e software (National Institutes of Health, Bethesda, MD, USA).

After washing the RBCs (three times), 20 µL of erythrocytes were resuspended in 40 µL of saline solution, subsequently diluted 1:1000 (*v*:*v*) in the solution for Sysmex blood analyzer (Sysmex F-820, Kobe, Japan) and filtered through the hydrophilic PVDF filter with 5 µm pores in diameter (Ultrafree-MC SV Centrifugal Filter, Merck Milipore Ltd., Carrigtwohill, Ireland) by centrifugation at 175× *g* during 5 min at room temperature. The deformability of RBCs was calculated as a percentage of filtered erythrocytes from the number of erythrocytes counted before centrifugation as described previously [22,23]. The intra sample variability of this method was about 5–10%. Mean cell volume (MCV) was measured by the Sysmex blood analyzer, while its value before the filtration was taken into consideration.

### 2.5. Statistical Analyses

The normality of data was analyzed by D’Agostino–Pearson test. Outliers were detected using the Grubbs’ test and subsequently removed from further analyses. The data are presented as mean ± standard deviations (SD). Statistical significance was analyzed by 2-way ANOVA for the main factors: age and rat strain, i.e., the effect of age and rat strain (i.e., hypertension), as well as their interaction (to understand whether the effect of age on the dependent variable is the same for both rat strains, and vice versa—to understand whether the effect of rat strain on the dependent variable is the same for all age categories) was analyzed. In the case of nonparametric data, we worked with log-transformed data to induce the normal distribution necessary for ANOVA analysis. Following the 2-way ANOVA, Tukey’s multiple comparisons post hoc test was applied to reveal differences between the individual experimental groups. To explore the relationships between variables, Pearson or Spearman correlation coefficients were calculated, depending on the normality of data distribution. Values were considered significant at *p* < 0.05., GraphPad Prism 7.02 (GraphPad Software, Inc., San Diego, CA, USA) was used for all the abovementioned analyses.

## 3. Results

### 3.1. Basic Biometric and Cardiovascular Characteristics of Experimental Animals

**Figure 1:**
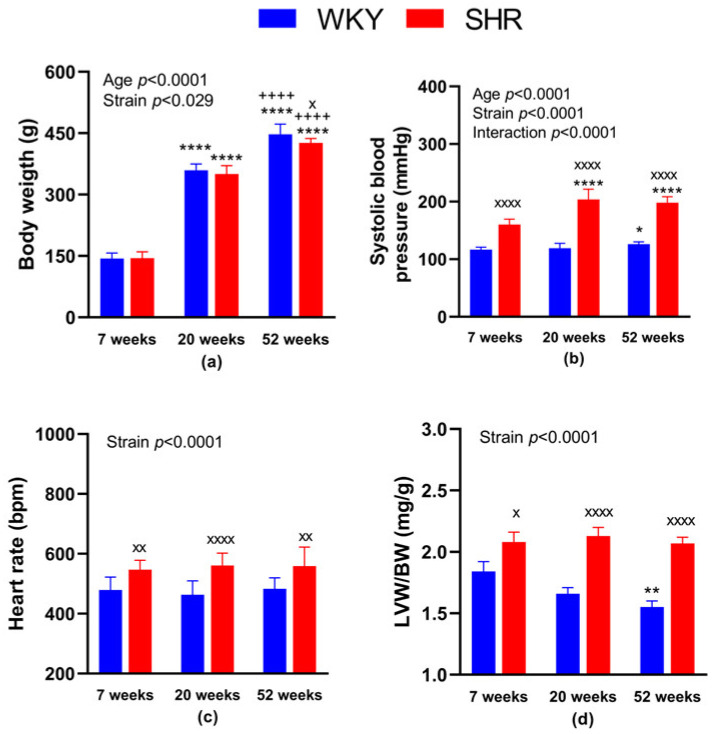
General characteristics of experimental animals: body weight (**a**), systolic blood pressure (**b**), heart rate (**c**) and the weight of the heart left ventricle adjusted to body weight (**d**). The data are presented as means ± standard deviations. Abbreviations: WKY—normotensive Wistar-Kyoto rats, SHR—spontaneously hypertensive rats, LVW—left ventricular weight, BW—body weight. Statistical significance: * *p* < 0.05, ** *p* < 0.01, **** *p* < 0.0001 vs. 7-week-old rat of the same strain; ^++++^ *p* < 0.0001 versus 20-week-old rat of the same strain; ^x^ *p* < 0.05, ^xx^ *p* < 0.01, ^xxxx^ *p* < 0.0001 vs. WKY rat of the same age. The number of animals in individual groups: WKY-7: *n* = 10, WKY-20: *n* = 9, WKY-52: *n* = 9, SHR-7: *n* = 10–11, SHR-20: *n* = 10 and SHR-52: *n* = 12.

Regarding the BW, it was lower in SHR-52 compared to age-matched WKY, while it was not changed between WKY and SHR strains in 7- and 20-week-old animals. Focusing on the age of both strains, BW was significantly lower in 7-week-old animals than in 20 and 52-week-old animals, and it was also lower in 20-week-old animals than in 52-week-old animals (Figure 1a). BP was significantly higher in all SHR groups compared with age-matched WKY animals. Focusing on WKY groups separately, BP was higher in WKY-52 than in WKY-7. Regarding the SHR groups, BP was lower in SHR-7 than in SHR-20 and SHR-52 (Figure 1b). Heart rate was increased in all SHRs compared with age-matched WKY rats (Figure 1c). The normalized weight of the left heart ventricle (i.e., adjusted to BW) was consistently higher in the SHRs compared with age-matched WKY rats. In addition, WKY-52 showed a lower LVW/BW ratio in comparison with WKY-7 rats (Figure 1d).

### 3.2. Biochemical Analysis of Blood Plasma

A summary of measured parameters with the statistical analysis is available in Table 1. For the concentration of the NT-proBNP, the strain factor was found to be significant (*p* = 0.0004) in a two-way ANOVA statistical tool. NT-proBNP was highest in SHR-7, being significantly different compared with age-matched normotensive counterparts.

Regarding the blood lipids, plasma cholesterol concentration showed significance for both factors—strain and age, as well as their interaction (*p* < 0.0001 for all). The total cholesterol was lower in SHR-20 and SHR-52 in comparison with the age-matched WKY rats, as well as in SHR-20 and SHR-52 in comparison with the SHR-7 group. In WKY-52, the total cholesterol was higher than in 7 and 20-week-old animals. For the concentration of triglycerides, age factor (*p* = 0.0038) and interaction between the age and strain (*p* = 0.0007) reached the level of statistical significance, while it was greater in SHR-7 when compared with age-matched normotensive counterparts, as well as when compared with older strain-matched animals, i.e., SHR-20 and SHR-52. Uric acid levels were lower in the plasma of SHR-20 and SHR-52 than in SHR-7 animals with a significant age factor (*p* = 0.011). We found a significant negative correlation between UA concentration and systolic BP value in SHRs (r = −0.58, *p* = 0.023), but not in WKY rats (r = −0.20, *p* = 0.471). The concentration of bilirubin was greater in WKY-20 and WKY-52 than in WKY-7. Plasma bilirubin level was higher in older and in normotensive rats with a significant age factor (*p* = 0.002), as well as strain factor (*p* = 0.0001). Regarding the oxidative stress markers, there was a statistically significant interaction between the effects of strain and age on GSH/GSSG ratio (*p* = 0.0002) and TBARS concentration (*p* < 0.0001). The effect of age was significant regarding the concentration of AGEs, fructosamine, TBARS and FRAP, while the strain factor showed significance in TBARS and fructosamine concentrations. Results of the multiple comparison tests are available in Table 1. The concentration of AGEs negatively correlated with BW in WKY (r = −0.67, *p* = 0.0004) as well as in all animals (r = −0.48, *p* = 0.0009), but not in SHRs (r = −0.17, *p* = 0.4540).

### 3.3. Erythrocyte Characteristics

#### 3.3.1. RBC Deformability—Effect of Age

We found significantly reduced RBC deformability in 52-week-old animals in both SHR and WKY as compared with strain-matched 20-week-old animals (in %: 59.6 ± 7.0 in SHR-52 vs. 78.5 ± 2.5 in SHR-20, *p* < 0.0001; 51.2 ± 7.1 in WKY-52 vs. 75.8 ± 4.4 in WKY-20, *p* < 0.0001). RBC deformability in 20-week-old animals in both groups (SHR and WKY) was higher in comparison with strain-matched 7-week-old animals (in %: 78.5 ± 2.5 in SHR-20 vs. 62.0 ± 6.9 in SHR-7, *p* < 0.0001; 75.8 ± 4.4 in WKY-20 vs. 68.9 ± 4.5 in WKY-7, *p* = 0.04). Comparing the 52- and 7-week-old animals, we found lower RBC deformability in WKY-52 (in %: 51.2 ± 7.1 in WKY-52 vs. 68.9 ± 4.5 in WKY-7, *p* < 0.0001), but no difference in SHR strain (in %: 59.6 ± 7.0 in SHR-52 vs. 62.0 ± 6.9 in SHR-7, *p* = 0.75).

#### 3.3.2. RBC Deformability—Effect of Hypertension

**Figure 2:**
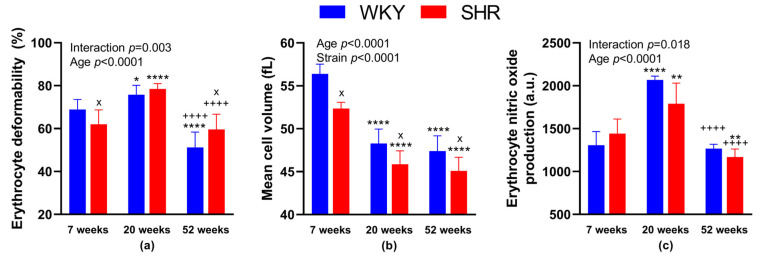
Erythrocyte parameters: deformability (**a**), mean cell volume (**b**) and nitric oxide production (**c**). The data are presented as means ± standard deviations. Abbreviations: WKY—normotensive Wistar-Kyoto rats, SHR—spontaneously hypertensive rats, a.u.—arbitrary units. Statistical significance: * *p* < 0.05, ** *p* < 0.01, **** *p* < 0.0001 vs. 7-week-old rat of the same strain; ^++++^ *p* < 0.0001 vs. 20-week-old rat of the same strain; ^x^ *p* < 0.05 vs. WKY rat of the same age. The number of animals in individual groups: WKY-7: *n* = 5–9, WKY-20: *n* = 5–8, WKY-52: *n* = 5–7, SHR-7: *n* = 5–8, SHR-20: *n* = 5–8, and SHR-52: *n* = 8.

Statistically significant strain-related differences in RBC deformability were observed at 7 and 52 weeks of age, where SHR-7 had reduced deformability (in %: 62.0 ± 6.9 in SHR-7 vs. 68.9 ± 4.5 in WKY-7, *p* = 0.038) and SHR-52 had increased RBC deformability as compared with age-matched WKY (in %: 59.6 ± 7.0 in SHR-52 vs. 51.2 ± 7.1 in WKY-52, *p* = 0.017). The summary of measurements is presented in Figure 2a.

**Figure 3:**
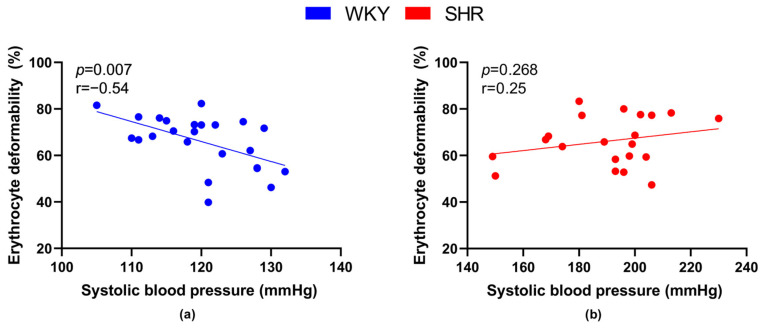
Relations between erythrocyte deformability and systolic blood pressure values in normotensive Wistar-Kyoto rats (WKY, *n* = 23) (**a**) and spontaneously hypertensive rats (SHR, *n* = 21) (**b**)—in both strains of rats, animals aged 7, 20 and 52 weeks were included.

We also focused on the relationship between RBC deformability and the value of systolic BP. A significant negative correlation between BP and deformability was observed in WKY (Figure 3a), but not in SHRs (Figure 3b).

#### 3.3.3. The Size of RBCs

Comparing the different strains—WKY and age-matched SHR, MCV was lower in SHRs of all age categories (in fL: 56.4 ± 1.1 in WKY-7 vs. 52.4 ± 0.7 in SHR-7, *p* = 0.01; 48.3 ± 1.7 in WKY-20 vs. 45.9 ± 1.6 in SHR-20, *p* = 0.01; 47.4 ± 1.8 in WKY-52 vs. 45.1 ± 1.6 in SHR-52, *p* = 0.02). Comparing the different ages of rats in individual strains, MCV was higher in WKY-7 than in WKY-20 and WKY-52. Similarly, MCV was higher in SHR-7 than in SHR-20 and SHR-52. The difference between 20- and 52-week-old animals of both strains was not observed (Figure 2b).

**Figure 4:**
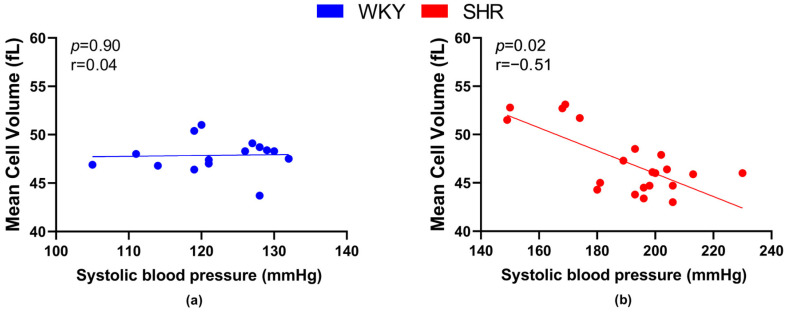
Relations between mean cell volume and systolic blood pressure values in normotensive Wistar-Kyoto rats (WKY, *n* = 15) (**a**) and spontaneously hypertensive rats (SHR, *n* = 21) (**b**)—in both strains of rats, animals aged 7, 20 and 52 weeks were included.

Regarding the relation between the size of RBCs and BP, a significant negative correlation was found in SHRs (Figure 4b), but not in WKY rats (Figure 4a).

#### 3.3.4. NO Production by RBCs—Effect of Age

NO production in RBCs was higher in WKY-20 and SHR-20 when compared with 7-week-old rats of the same phenotype (in a.u.: 2067 ± 45 in WKY-20 vs. 1308 ± 158 in WKY-7, *p* < 0.0001; 1790 ± 240 in SHR-20 vs. 1443 ± 169 in SHR-7, *p* = 0.001). In SHR-52, we found a significant decrease in NO production in comparison with SHR-7 (in a.u.: 1169 ± 93 in SHR-52 vs. 1443 ± 169 in SHR-7, *p* = 0.003). In addition, we found statistically significant reductions in NO production at 52-week-old animals in both, SHR and WKY strains when compared with the respective 20-week-old group of strain-matched rats (in a.u.: 1169 ± 93 in SHR-52 vs. 1790 ± 240 in SHR-20, *p* < 0.0001; 1267 ± 51 in WKY-52 vs. 2067 ± 45 in WKY-20, *p* < 0.0001).

#### 3.3.5. NO Production by RBCs—Effect of Hypertension

We observed no statistical differences comparing both WKY and SHR age-matched animals, only a tendency to lower NO production in SHR-20 when compared with WKY-20 (*p* = 0.05). The summary of measurements is presented in Figure 2c.

Regarding the relation between the NO production by RBCs and systolic BP values, no significant correlations were found.

#### 3.3.6. Relations between RBC Parameters in Normotensive and Hypertensive Rats

**Figure 5:**
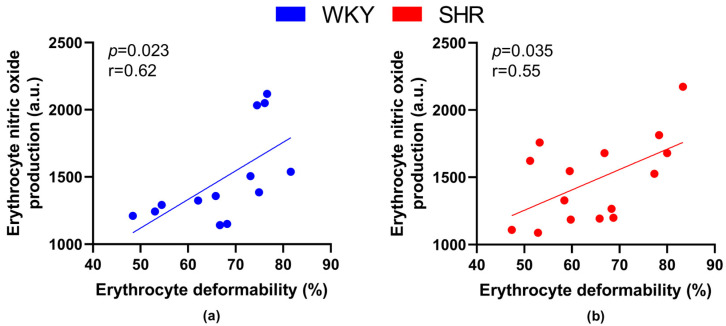
Relations between erythrocyte deformability and nitric oxide production by erythrocytes in normotensive Wistar-Kyoto (WKY, *n* = 13) (**a**) rats and spontaneously hypertensive rats (SHR, *n* = 15) (**b**)—in both strains of rats, animals aged 7, 20 and 52 weeks were included.

When focusing on relations between RBC parameters—deformability, MCV and NO production, RBC deformability positively correlated with NO production by RBCs in all animals (r = 0.56, *p* = 0.002), as well as in all WKY (r = 0.62, *p* = 0.023) and all SHR (r = 0.546, *p* = 0.035) separately (Figure 5).

Correlating the RBC deformability and NO production in RBCs in individual groups, we found a clear tendency towards a statistically significant positive correlation in WKY-52 (r = 0.95, *p* = 0.05). A certain trend toward similar significance was observable within the remaining individual experimental groups (r = 0.44–0.92), but it failed to achieve that threshold value for *p* (most probably due to the low count of animals in individual groups).

**Figure 6:**
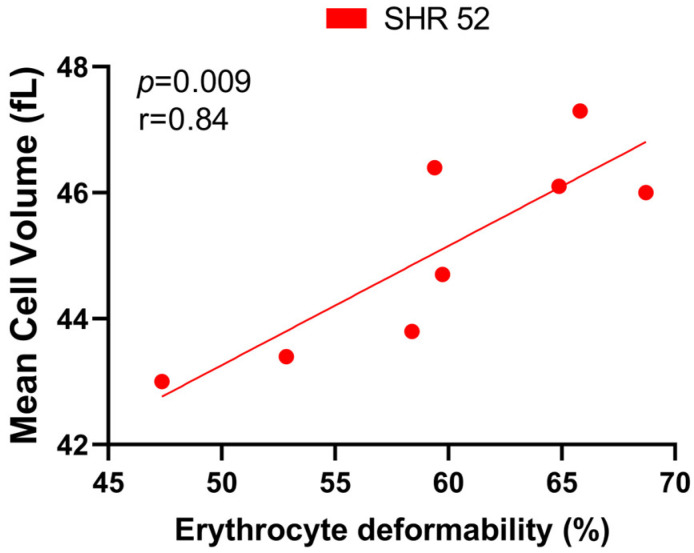
Relationship between erythrocyte deformability and mean cell volume in spontaneously hypertensive rats (SHRs) at 52 weeks of age (*n* = 8).

Correlating the RBC deformability and MCV, we found a statistically significant positive correlation only in SHR-52 (r = 0.84, *p* = 0.009, Figure 6). However, when focusing on other individual experimental groups or their combinations, no tendency for a correlation between RBC deformability and MCV was found.

Correlating the MCV and NO production by RBCs, we found no correlation within experimental groups.

#### 3.3.7. Correlations of RBC Parameters and Biochemical Parameters in Blood Plasma

**Figure 7:**
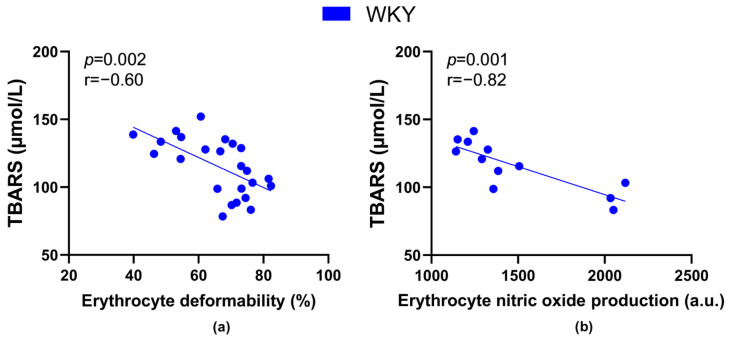
Relations between the plasma concentration of markers of lipid peroxidation-thiobarbituric acid reactive substances (TBARS) and erythrocyte deformability ((**a**), *n* = 24) as well as nitric oxide production by erythrocytes ((**b**), *n* = 12) in Wistar-Kyoto rats (WKY)—animals aged 7, 20 and 52 weeks were included.

The TBARS levels negatively correlated with values of RBC deformability (Figure 7a) as well as NO production by RBCs (Figure 7b) in all WKY rats, but not in SHRs.

**Figure 8:**
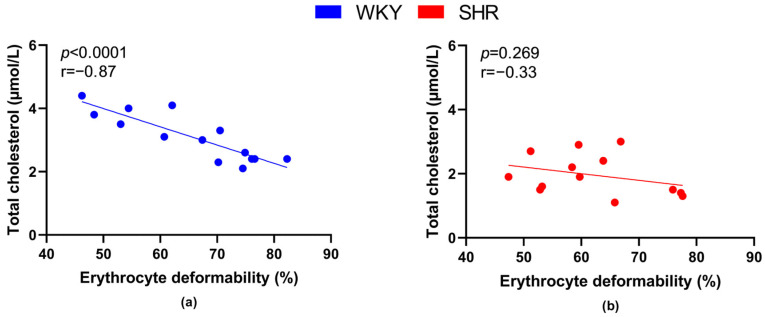
Relations between erythrocyte deformability and plasma levels of total cholesterol in normotensive Wistar-Kyoto rats (WKY, *n* = 14) (**a**) and spontaneously hypertensive rats (SHR, *n* = 13) (**b**)—in both strains of rats, animals aged 7, 20 and 52 weeks were included.

Plasma cholesterol concentration was inversely proportional to RBC deformability in all rats (r = −0.39, *p* = 0.043), as well as in all WKY rats (Figure 8a), but not in SHRs (Figure 8b).

## 4. Discussion

Using WKY rats and SHRs of three different age categories—young juvenile (7 weeks), young adult (20 weeks) and older (52 weeks), we managed to obtain more detailed information about the changes in selected properties of RBCs occurring during aging in normotensive and hypertensive individuals.

### 4.1. SHRs—The Model of Genetic Hypertension, Their Normotensive Controls, and Aging

Regarding the basic characteristics of experimental animals, the results of our study confirmed some already known information about SHRs and their normotensive WKY counterparts. According to other papers, WKY and SHR showed similar patterns of BW gain until 16 weeks of age [25], while above 12 weeks of age, BW was shown to be significantly higher in Wistar rats than in SHR [26], although this was not observed in a previously published study [27]. Our study supports the observation of lower age-related BW increase in SHR strain as shown by the strain-related difference in BW of 52-week-old animals.

As expected, systolic BP was higher in SHR than that in age-matched WKY, but we have also noted age-related BP increase in WKY-52 as compared with younger animals (although not reaching the values for hypertension), and likewise in human populations during aging, most probably due to an age-related increase in the arterial stiffness. In SHR, the rise in systolic BP was evident until 20 weeks of age; afterwards, it remained stable, which is in line with previously published data [28,29]. Consistent with previous observations [30], heart rate and normalized left ventricular mass were observed to be greater in SHRs than in normotensive rats regardless of age. The LVW/BW ratio decreased with aging in normotensive rats which was also previously observed in humans, suggesting a decrease in cardiomyocyte mass during the aging process [31], but not in the condition of hypertension.

Focusing on changes in NT-proBNP, our data confirmed its higher plasma concentrations in SHRs compared with WKY rats in general. However, we also expected an age-related increase in NT-proBNP concentration, especially in hypertensive animals. Since this was not observed, it can be assumed that this may be a consequence of neurohumoral adaptation to high BP after its stabilization in the used model of essential hypertension. In this context, it is also worth adding that our older hypertensive animals did not show signs of congestive heart failure. The lack of differences between normotensive and hypertensive animals in the 20-week and 52-week age groups could also be attributed to the large amount of variation in plasma NT-proBNP levels in SHRs, as indicated by the relatively higher SD values.

### 4.2. RBC Deformability and NO Production by RBCs—Focus on Age-Related Changes

The effect of age was manifested in 20-week-old WKY rats as well as in SHRs, occurring concomitantly with a significant increase in RBC deformability and NO production when compared with 7-week-old rats of the same strain. Relatively few studies have focused on potential changes in these parameters that may occur between childhood and adulthood. The elastic modulus of RBC membrane was measured in WKY and SHR over a 20-week period, i.e., from 3 to 23 weeks of age, while fluctuations in RBC membrane rigidity were found in both rat strains [32]. Our data are more similar to those obtained by a study focused on the period from birth to 3 months of age in Wistar rats, where an increase in RBC deformability index was observed up to 4 weeks of age, while its decrease was observed later [33]. To the best of our knowledge, the changes in NO production by RBCs between the young juvenile and young adult individuals were not documented yet. Therefore, more studies are needed to precisely understand the dynamics of RBC viscoelastic properties in the early period of life.

Furthermore, we observed statistically significant reductions in RBC deformability as well as NO production at 52 weeks in both, SHR and WKY strains when compared with the strain-matched 20-week-old rats. A similar age-related decrease in NO production by RBCs was also observed previously in 6- and 12-month-old lean and obese Zucker diabetic fatty rats [34]. Age-related deterioration of RBC deformability or decrease in the RBC membrane fluidity was also observed in humans [2,35,36]. Interestingly, the age-related decline in RBC deformability, as well as in NO production by RBCs, was greater in normotensive animals than the hypertensive ones in our experiment.

Taken together, our results support the idea that RBC deformability and NO production by RBCs could still increase at younger age, while there is a decrease in both parameters at an older age. Thus, we can presume that age-related changes in RBC deformability of both WKY and SHR are at least partially associated with changes in NO production by RBCs. NO produced by RBCs has been documented to react with cytoskeletal proteins (α- and β-spectrins), forming S-nitrosothiols and promoting RBC deformability [37]. Furthermore, such post-translational modification protects the RBC membrane from potential oxidative damage [38].

### 4.3. RBC Deformability and NO Production by RBCs—Focus on Strain Differences (Normotension versus Hypertension)

The results of our study showed significant changes in RBC deformability in SHR-7 and SHR-52 rats in comparison to age-matched WKY rats. RBC deformability was found to be lower in the condition of hypertension according to numerous studies (for review, see [2]). However, this trend was observable only in young juvenile (7 weeks old) animals in our study. We can hypothesize that the decrease in RBC deformability is an early phenomenon in SHRs. In 20-week-old rats, there was no difference between RBC deformability in normotensive WKY rats and SHRs, suggesting an occurrence of an adaptive recovery and promotion of RBC deformability in adult age. A similar tendency was observed in the study of Chabanel et al. [32], in which RBC membrane elasticity was investigated in WKY and SHR ranging from 3 to 23 weeks of age, while an increase in RBC membrane rigidity was observed prior the definitive establishment of hypertension. As RBC deformability was higher in SHR compared with WKY rats in older (i.e., in 52-week-old) animals, one may presume that it may represent an adaptation to prolonged hypertension; however, this was not reported by human studies. Thus, experiments dealing with erythrocyte properties in older hypertensive rats may not be directly approximated to the situation in human hypertension.

Nevertheless, it is unlikely that changes in RBC deformability observed between normo- and hypertensive individuals are related to changes in NO production by RBCs. The mechanism responsible for better RBC deformability in SHR-52 when compared with WKY-52 requires further investigation.

### 4.4. The Size of RBCs—Strain and Age-Related Changes

Regarding the size of RBCs that is commonly estimated by determining the MCV, our data are in line with the reported decrease in MCV during aging in the Wistar, WKY, SHR and Sprague Dawley rats [32,39,40,41]; however, the opposite, i.e., an annual increase in MCV value, was observed in humans [42,43,44,45]. This may represent further limitation when data obtained by the use of experimental animals are attempted to be applied to humans.

Hypertension in rats, as well as in humans, is accompanied by smaller RBCs (in comparison with those from normotensives) according to our results as well as previously published data [28,32,46], while MCV value was identified to be inversely proportional to systolic BP value [47]. It is just speculation that a lower volume of RBCs may represent a compensatory mechanism to reduce the resistance to blood flow in the condition of hypertension. It can also be a consequence of impaired transport of cations across the RBC membrane [2,44,46]. In general, multiple ion channels are involved in RBC volume control [48]. In addition to ATP-driven transporters, there are passive transport systems, also called gradient-driven transporters, in RBC membranes. One of them, the Gardos channel, is responsible for the potassium efflux in response to an increase in the intracellular calcium concentration. Activation of the Gardos channel results in RBC shrinkage [49]. In hypertension, calcium ion entry may be promoted by the activation of mechanosensitive PIEZO1 channels, thereby leading to the decrease in MCV observed in our hypertensive rats. This hypothesis is consistent with the previous observation of increased Ca^2+^-dependent K^+^ efflux documented in RBCs from hypertensive patients [50]. However, a decrease in MCV does not promote RBC deformability in our experimental animals. The lower MCV value was also observed in older RBCs in the previous study [51], so one can assume that the portion of older RBCs in SHRs is higher than in normotensive individuals.

### 4.5. Relations between Blood Pressure Value and Erythrocyte Parameters

In contrast with hypertensive rats, RBC deformability was inversely proportional to the values of systolic BP in normotensive animals included in our study. An improvement in RBC deformability was observed to be associated with the promotion of whole blood viscosity in healthy individuals [24] which can represent the mechanism responsible for lowering the BP. However, a direct relation between BP value and whole blood viscosity was observed not only in normotensive, but also in hypertensive individuals [52]. Given the absence of a relationship between RBC deformability and systolic BP value in our hypertensive animals, changes in blood plasma composition could be more responsible for the blood pressure elevation in the hypertensive state that neutralizes the expected effect resulting from changes in RBC properties. This hypothesis is consistent with the previously observed higher plasma viscosity in SHRs [53].

### 4.6. What Can Be Responsible for Differences in RBC Deformability in Normotension and Hypertension?

In normotensive and hypertensive animals, we observed a direct relationship between RBC deformability and NO production by RBCs—indicating a beneficial effect of NO produced by RBCs on their ability to change their shape in both conditions. This was already observed previously in humans, as well as in experimental animals [38]. It should be noted that the aging of an individual does not have the same consequences on RBC properties as the aging of RBCs themselves (i.e., during their lifetime), as the highest NO production was observed in the old RBCs that were also less deformable [51]. Nevertheless, NO production by RBCs appears to affect RBC deformability during aging in the same way in normotensive as well as hypertensive individuals.

RBCs from hypertensives were shown to be more affected by oxidative stress as indicated by a higher count of carbonyl groups in the RBC membranes of these patients [54]. However, our oxidative stress markers do not confirm a significant deterioration of either oxidative or carbonyl stress in our animals during aging as discussed previously [20]. The fact that oxidative stress is not primarily responsible for changes in RBC deformability is also supported by the fact that lipid peroxidation detected in blood plasma was inversely proportional to deformability as well as NO production by RBCs only in normotensive animals.

An increase in serum cholesterol level was linked with a decrease in RBC membrane deformability while statin administration improved it [2,55]. These observations are in line with a significant inverse relation between RBC deformability and plasma cholesterol concentration detected in our normotensive WKY rats. However, lower cholesterol levels are typical for SHRs, as shown by our results as well as previously published studies [56,57,58], and interestingly, the negative relationship between plasma cholesterol and RBC deformability did not reach a level of statistical significance indicating a potential limitation of the primary effect of plasma cholesterol on RBC deformability in hypertension.

In addition to the variability of the presented results that makes it difficult to identify the primary factor unequivocally responsible for changes in RBC deformability in the condition of hypertension, another factor can also be taken into consideration. As shown previously, an increase in BP was accompanied by the accumulation of membrane tubulin in RBCs of SHRs at 42 days of age [6] which could explain the reduction in RBC deformability in our hypertensive rats (compared with normotensive rats) observed at 7 weeks of life. With increasing age, deformability in SHRs relatively improved as no difference between WKY and SHRs at 20 weeks of age was observed, and furthermore, it was relatively better in older animals at 52 weeks of age. Reducing the size of RBCs does not promote their deformability. Noteworthy, in the group of SHRs with the smallest RBCs, i.e., in SHRs of 52 weeks of age, the size of RBCs positively correlated with RBC deformability.

In general, erythrocyte deformability is determined by following three factors: surface area per volume ratio, cytoplasmic rheology and cell membrane fluidity, while worsened ion transport through the RBC membrane in the condition of hypertension is responsible for the deterioration of RBC geometry, as well as internal viscosity [2,48,50]. A decrease in RBC membrane fluidity was observed in hypertensive animals and patients that was ascribed to changes in the lipid composition of RBC membranes [59,60]. Such changes involve higher phosphatidylcholine, phosphatidylethanolamine and phosphatidylserine contents and a lower cholesterol content in RBCs from hypertensives compared with those with normal BP [60]. It was also shown that RBCs of WKY and SHRs exhibit membrane structure differences existing already in young animals, i.e., prior the BP increase [61]. However, further research is needed to fully understand the changes in all determinants of RBC deformability during aging.

## 5. Conclusions

Summarizing our results, we can conclude that changes in NO production by RBCs under hypertensive conditions are an unlikely primary factor affecting RBC deformability, whereas age-related changes in RBC deformability of both WKY and SHRs are at least partially associated with changes in NO production. However, the interpretation of data obtained in RBC parameters observed in SHRs to human hypertension requires precaution.

## Figures and Tables

**Table 1:** Blood plasma analysis in rats of three age categories: 7-, 20- and 52-week-old animals.

	WKY-7 *n* = 5–12			SHR-7 *n* = 5–7			WKY-20 *n* = 6–10			SHR-20 *n* = 5–7			WKY-52 *n* = 5–7			SHR-52 *n* = 5–10		
NT-proBNP (pg/mL)	113	±	5.7	467	±	160 ^xx^	148	±	44	230	±	163	171	±	70	294	±	187
Total cholesterol (mmol/L)	2.6	±	0.42	2.9	±	0.34	2.8	±	0.53	1.4	±	0.11 *^,x^	4.0	±	0.34 *^,+^	1.7	±	0.43 *^,x^
Triglycerides (mmol/L)	1.3	±	0.33	1.9	±	0.28 ^x^	1.4	±	0.21	1.3	±	0.32 *	1.3	±	0.23	0.93	±	0.55 *
Uric acid (µmol/L)	40	±	11	53	±	18	45	±	16	35	±	9.4 *	29	±	13 ^+^	30	±	9.2 *
Bilirubin (µmol/L)	1.4	±	0.68	0.82	±	0.18	2.2	±	0.55 ***	1.2	±	0.29 ^xx^	2.1	±	0.084 *	1.5	±	0.51
GSH/GSSG	11	±	0.85	17	±	3.9 ^xx^	17	±	1.5 **	13	±	1.3	15	±	3.9	17	±	3.8
TBARS (µmol/L)	120	±	22	127	±	16	95	±	8.4	71	±	7.5 ***	132	±	7.8 ^++^	58	±	13 ***^,xxx^
FRUC (mmol/g)	17	±	4.5	21	±	3.5	16	±	3.7	18	±	6.1	11	±	2.3	15	±	5.0
AGEs (mg/g)	19	±	4.2	19	±	5.1	12	±	1.9 **	9.7	±	1.1 ***	12	±	2.1 ***	15	±	1.8 ^+^
FRAP (mmol/L)	1.2	±	0.15	1.3	±	0.23	1.0	±	0.14	1.1	±	0.12	1.1	±	0.17	1.1	±	0.10
AOPP (µmol/g)	2.3	±	0.87	3.5	±	1.00	2.9	±	0.89	2.6	±	0.83	2.01	±	0.64	2.1	±	0.81

The data are presented as means ± standard deviations. Abbreviations: WKY—Wistar-Kyoto rats, SHR—spontaneously hypertensive rats, NT-proBNP—N-terminal pro-brain natriuretic peptide, GSH/GSSG—the ratio of reduced to oxidized glutathione, TBARS—thiobarbituric acid reactive substances, FRUC—fructosamine, AGEs—advanced glycation end products, FRAP—ferric reducing antioxidant power, AOPP—advanced oxidation protein products. Statistical significance: * *p* < 0.05, ** *p* < 0.01, *** *p* < 0.001 vs. 7-week-old rat of the same strain; ^+^ *p* < 0.05, ^++^ *p* < 0.01 vs. 20-week-old rat of the same strain; ^x^ *p* < 0.05, ^xx^ *p* < 0.01, ^xxx^ *p* < 0.001 versus WKY rat of the same age.

## Data Availability

The data presented in this study are available on request from the corresponding author.

## References

[1] Shin S., Ku Y., Park M.-S., Suh J.-S. (2005). Deformability of red blood cells: A determinant of blood viscosity. J. Mech. Sci. Technol..

[2] Radosinska J., Vrbjar N. (2016). The Role of Red Blood Cell Deformability and Na,K-ATPase Function in Selected Risk Factors of Cardiovascular Diseases in Humans: Focus on Hypertension, Diabetes Mellitus and Hypercholesterolemia. Physiol. Res..

[3] Radosinska J., Vrbjar N. (2021). Erythrocyte Deformability and Na,K-ATPase Activity in Various Pathophysiological Situations and Their Protection by Selected Nutritional Antioxidants in Humans. Int. J. Mol. Sci..

[4] Cicco G., Vicenti P., Stingi G.D., Tarallo M.S., Pirrelli A. (1999). Hemorheology in complicated hypertension. Clin. Hemorheol. Microcirc..

[5] Cicco G., Pirrelli A. (1999). Red blood cell (RBC) deformability, RBC aggregability and tissue oxygenation in hypertension. Clin. Hemorheol. Microcirc..

[6] Amaiden M.R., Monesterolo N.E., Santander V.S., Campetelli A.N., Arce C.A., Pie J., Hope S.I., Vatta M.S., Casale C.H. (2012). Involvement of membrane tubulin in erythrocyte deformability and blood pressure. J. Hypertens..

[7] Plotnikov M.B., Aliev O.I., Nosarev A.V., Shamanaev A.Y., Sidekhmenova A.V., Anfinogenova Y., Anishchenko A.M., Pushkina E.V. (2016). Relationship between arterial blood pressure and blood viscosity in spontaneously hypertensive rats treated with pentoxifylline. Biorheology.

[8] Shabanov V.A., Terekhina E.V., Kostrov V.A. (2001). Changes in blood rheological properties in patients with hypertension. Ter. Arkhiv.

[9] Fu G.-X., Ji M., Han L.-Z., Xu C.-C., Pan F.-F., Hu T.-J., Zhong Y. (2017). Erythrocyte rheological properties but not whole blood and plasma viscosity are associated with severity of hypertension in older people. Z. Gerontol. Geriatr..

[10] Michalska-Małecka K., Słowińska-Łożyńska L. (2012). Aggregation and deformability of erythrocytes in primary open-angle glaucoma (POAG); the assessment of arterial hypertension. Clin. Hemorheol. Microcirc..

[11] Bor-Kucukatay M., Wenby R.B., Meiselman H.J., Baskurt O.K. (2003). Effects of nitric oxide on red blood cell deformability. Am. J. Physiol. Circ. Physiol..

[12] Cortese-Krott M.M., Kelm M. (2014). Endothelial nitric oxide synthase in red blood cells: Key to a new erythrocrine function?. Redox Biol..

[13] Nava E., Farré A.L., Moreno C., Casado S., Moreau P., Cosentino F., Lüscher T.F. (1998). Alterations to the nitric oxide pathway in the spontaneously hypertensive rat. J. Hypertens..

[14] Tegeder I. (2019). Nitric oxide mediated redox regulation of protein homeostasis. Cell. Signal..

[15] Buford T.W. (2016). Hypertension and aging. Ageing Res. Rev..

[16] Dickhout J.G., Lee R.M.K.W. (1998). Blood pressure and heart rate development in young spontaneously hypertensive rats. Am. J. Physiol. Circ. Physiol..

[17] Dornas W.C., Silva M.E. (2011). Animal models for the study of arterial hypertension. J. Biosci..

[18] Sagvolden T. (2000). Behavioral validation of the spontaneously hypertensive rat (SHR) as an animal model of attention-deficit/hyperactivity disorder (AD/HD). Neurosci. Biobehav. Rev..

[19] Kluknavsky M., Balis P., Puzserova A., Radosinska J., Berenyiova A., Drobna M., Lukac S., Muchova J., Bernatova I. (2016). (−)-Epicatechin Prevents Blood Pressure Increase and Reduces Locomotor Hyperactivity in Young Spontaneously Hypertensive Rats. Oxid. Med. Cell. Longev..

[20] Kollarova M., Puzserova A., Balis P., Radosinska D., Tothova L., Bartekova M., Barancik M., Radosinska J. (2020). Age- and Phenotype-Dependent Changes in Circulating MMP-2 and MMP-9 Activities in Normotensive and Hypertensive Rats. Int. J. Mol. Sci..

[21] Berenyiova A., Balis P., Kluknavsky M., Bernatova I., Cacanyiova S., Puzserova A. (2022). Age- and Hypertension-Related Changes in NOS/NO/sGC-Derived Vasoactive Control of Rat Thoracic Aortae. Oxid. Med. Cell. Longev..

[22] Jasenovec T., Radosinska D., Kollarova M., Vrbjar N., Balis P., Trubacova S., Paulis L., Tothova L., Shawkatova I., Radosinska J. (2022). Monocrotaline-Induced Pulmonary Arterial Hypertension and Bosentan Treatment in Rats: Focus on Plasma and Erythrocyte Parameters. Pharmaceuticals.

[23] Jasenovec T., Radosinska D., Kollarova M., Balis P., Zorad S., Vrbjar N., Bernatova I., Cacanyiova S., Tothova L., Radosinska J. (2022). Effects of Taxifolin in Spontaneously Hypertensive Rats with a Focus on Erythrocyte Quality. Life.

[24] Radosinska J., Jasenovec T., Puzserova A., Krajcir J., Lacekova J., Kucerova K., Kalnovicova T., Tothova L., Kovacicova I., Vrbjar N. (2019). Promotion of whole blood rheology after vitamin C supplementation: Focus on red blood cells. Can. J. Physiol. Pharmacol..

[25] Park S., Shin J., Hong Y., Kim S., Lee S., Park K., Lkhagvasuren T., Lee S.-R., Chang K.-T., Hong Y. (2012). Forced Exercise Enhances Functional Recovery after Focal Cerebral Ischemia in Spontaneously Hypertensive Rats. Brain Sci..

[26] Labat C., Cunha R.S.A., Challande P., Safar M.E., Lacolley P. (2006). Respective contribution of age, mean arterial pressure, and body weight on central arterial distensibility in SHR. Am. J. Physiol. Circ. Physiol..

[27] Niewiadomska G., Łukaszewska I. (1987). Increase in body weight of spontaneously hypertensive rats (SHR) under prolonged behavioral stimulation. Physiol. Behav..

[28] Bruschi G., Minari M., Bruschi M.E., Tacinelli L., Milani B., Cavatorta A., Borghetti A. (1986). Similarities of essential and spontaneous hypertension. Volume and number of blood cells. Hypertension.

[29] Boylan J.W., Van Liew J.B., Feig P.U. (1991). Inverse changes in erythroid cell volume and number regulate the hematocrit in newborn genetically hypertensive rats. Proc. Natl. Acad. Sci. USA.

[30] Naessens D.M.P., de Vos J., Richard E., Wilhelmus M.M.M., Jongenelen C.A.M., Scholl E.R., van der Wel N.N., Heijst J.A., Teunissen C.E., Strijkers G.J. (2023). Effect of long-term antihypertensive treatment on cerebrovascular structure and function in hypertensive rats. Sci. Rep..

[31] Melissari M., Balbi T., Gennari M., Olivetti G. (1991). The aging of the heart: Weight and structural changes in the left ventricle with age. G. Ital. Cardiol..

[32] Chabanel A., Schachter D., Chien S. (1987). Increased rigidity of red blood cell membrane in young spontaneously hypertensive rats. Hypertension.

[33] Novozhilov A.V., Katiukhin L.N., Feĭzullaev B.A. (2012). Dynamics of hematologic parameters and of the erythrocyte deformability index at the juvenal period of rats and guinea pigs. Zh. Evol. Biokhim. Fiziol..

[34] Jasenovec T., Radosinska D., Kollarova M., Balis P., Ferenczyova K., Kalocayova B., Bartekova M., Tothova L., Radosinska J. (2021). Beneficial Effect of Quercetin on Erythrocyte Properties in Type 2 Diabetic Rats. Molecules.

[35] Ward K.A., Baker C., Roebuck L., Wickline K., Schwartz R.W. (1991). Red blood cell deformability: Effect of age and smoking. Age.

[36] Goi G., Cazzola R., Tringali C., Massaccesi L., Volpe S.R., Rondanelli M., Ferrari E., Herrera C.B., Cestaro B., Lombardo A. (2005). Erythrocyte membrane alterations during ageing affect β-d-glucuronidase and neutral sialidase in elderly healthy subjects. Exp. Gerontol..

[37] Grau M., Pauly S., Ali J., Walpurgis K., Thevis M., Bloch W., Suhr F. (2013). RBC-NOS-Dependent S-Nitrosylation of Cytoskeletal Proteins Improves RBC Deformability. PLoS ONE.

[38] Kobayashi J., Ohtake K., Murata I., Sonoda K. (2022). Nitric oxide bioavailability for red blood cell deformability in the microcirculation: A review of recent progress. Nitric Oxide.

[39] Loftus T.J., Kannan K.B., Carter C.S., Plazas J.M., Mira J.C., Brakenridge S.C.M., Leeuwenburgh C., Efron P.A., Mohr A.M. (2018). Persistent injury-associated anemia and aging: Novel insights. J. Trauma Inj. Infect. Crit. Care.

[40] Giuliani A., Graldi G., Veronesi M., Previato A., Simoni M., Bergamini C., Berti G. (2000). Binding of anti-spectrin antibodies to red blood cells and vesiculation in various in vivo and in vitro ageing conditions in the rat. Exp. Gerontol..

[41] Somogyi V., Peto K., Deak A., Tanczos B., Nemeth N. (2018). Effects of aging and gender on micro-rheology of blood in 3 to 18 months old male and female Wistar (Crl:WI) rats. Biorheology.

[42] Lee J.Y., Choi H., Park J.W., Son B.R., Park J.H., Jang L.C., Gil Lee J. (2022). Age-related changes in mean corpuscular volumes in patients without anaemia: An analysis of large-volume data from a single institute. J. Cell. Mol. Med..

[43] Goldberg I., Cohen E., Gafter-Gvili A., Shochat T., Kugler E., Margalit I., Goldberg E., Raanani P., Krause I. (2023). A Longitudinal Assessment of the Natural Change in Haemoglobin, Haematocrit, and Mean Corpuscular Volume with Age. Acta Haematol..

[44] Khecuriani R., Lomsadze G., Arabuli M., Sanikidze T. (2010). Deformability of red blood cells and human aging. Georgian Med. News.

[45] Vayá A., Alis R., Romagnoli M., Pérez R., Bautista D., Alonso R., Laiz B. (2013). Rheological blood behavior is not only influenced by cardiovascular risk factors but also by aging itself. Research into 927 healthy Spanish Mediterranean subjects. Clin. Hemorheol. Microcirc..

[46] Postnov Y.V., Kravtsov G.M., Orlov S.N., Pokudin N.I., Postnov I.Y., Kotelevtsev Y.V. (1988). Effect of protein kinase C activation on cytoskeleton and cation transport in human erythrocytes. Reproduction of some membrane abnormalities revealed in essential hypertension. Hypertension.

[47] Sharp D.S., Curb J.D., Schatz I.J., Meiselman H.J., Fisher T.C., Burchfiel C.M., Rodriguez B.L., Yano K. (1996). Mean Red Cell Volume as a Correlate of Blood Pressure. Circulation.

[48] Brugnara C. (1997). Erythrocyte membrane transport physiology. Curr. Opin. Hematol..

[49] Föller M., Lang F. (2020). Ion Transport in Eryptosis, the Suicidal Death of Erythrocytes. Front. Cell Dev. Biol..

[50] Adragna N., Canessa M., Bize I., Solomon H., Tosteson D.C. (1981). Ki^+^-Na^+^_o_ Countertransport in Erythrocytes of Patients with Essential Hypertension. Clin. Sci..

[51] Bizjak D.A., Brinkmann C., Bloch W., Grau M. (2015). Increase in Red Blood Cell-Nitric Oxide Synthase Dependent Nitric Oxide Production during Red Blood Cell Aging in Health and Disease: A Study on Age Dependent Changes of Rheologic and Enzymatic Properties in Red Blood Cells. PLoS ONE.

[52] Letcher R.L., Chien S., Pickering T.G., Sealey J.E., Laragh J.H. (1981). Direct relationship between blood pressure and blood viscosity in normal and hypertensive subjects: Role of fibrinogen and concentration. Am. J. Med..

[53] Shamanaev A.Y., Aliev O.I., Anishchenko A.M., Sidehmenova A.V., Plotnikov M.B. (2017). Hemorheological effects of amlodipine in spontaneously hypertensive rats. Indian J. Pharmacol..

[54] Kumar N., Maurya P.K., Kant R., Rizvi S.I. (2016). (−)-Epicatechin in vitro ameliorates erythrocyte protein carbonyl content in hypertensive patients: Comparison with L-ascorbic acid. Arch. Physiol. Biochem..

[55] Broncel M., Bała A., Koter-Michalak M., Duchnowicz P., Wojsznis W., Chojnowska-Jezierska J. (2007). Physicochemical modifications induced by statins therapy on human erythrocytes membranes. Wiadomości Lek..

[56] Hanson M.G., Zahradka P., Taylor C.G. (2014). Lentil-based diets attenuate hypertension and large-artery remodelling in spontaneously hypertensive rats. Br. J. Nutr..

[57] Kitts D.D., Yuan Y.V., Godin D.V. (1998). Plasma and Lipoprotein Lipid Composition and Hepatic Antioxidant Status in Spontaneously Hypertensive (SHR) and Normotensive (WKY) Rats. Can. J. Physiol. Pharmacol..

[58] Júnior S.A.O., Okoshi K., Lima-Leopoldo A.P., Leopoldo A.S., Campos D.H., Martinez P.F., Okoshi M.P., Padovani C.R., Pai-Silva M.D., Cicogna A.C. (2009). Nutritional and Cardiovascular Profiles of Normotensive and Hypertensive Rats Kept on a High Fat Diet. Arq. Bras. Cardiol..

[59] Nikelshparg E.I., Baizhumanov A.A., Bochkova Z.V., Novikov S.M., Yakubovsky D.I., Arsenin A.V., Volkov V.S., Goodilin E.A., Semenova A.A., Sosnovtseva O. (2022). Detection of Hypertension-Induced Changes in Erythrocytes by SERS Nanosensors. Biosensors.

[60] Martínez-Vieyra V., Rodríguez-Varela M., García-Rubio D., De La Mora-Mojica B., Méndez-Méndez J., Durán-Álvarez C., Cerecedo D. (2019). Alterations to plasma membrane lipid contents affect the biophysical properties of erythrocytes from individuals with hypertension. Biochim. Biophys. Acta BBA Biomembr..

[61] Montenay-Garestier T., Aragon I., Devynck M.-A., Meyer P., Helene C. (1981). Evidence for structural changes in erythrocyte membranes of spontaneously hypertensive rats. A fluorescence polarization study. Biochem. Biophys. Res. Commun..

